# Supplementary material for: Coordinated control of senescence by lncRNA and a novel T-box3 co-repressor complex
Source: eLife. 2014 May 29;3:e02805. doi: 10.7554/eLife.02805 (PMC4071561; doi:10.7554/eLife.02805)
Supplement: Figure 7—source data 3. — DOI: http://dx.doi.org/10.7554/eLife.02805.029 [file elife02805s003.docx]

**Supplemental Table 3**

**The hypergeometric test, as implemented in the R statistical language (phyper).**

# Total Number of TBX3/CAPER co-regulated genes: 1157

# Total Number of TBX3 regulated genes: 2188

# Number of non-TBX3 regulated genes: 22778

# Total Number of CAPER regulated genes: 2375

> phyper(1157, 2188, 22778, 2375, lower.tail=FALSE)

[1] 0

> phyper(1157, 2188, 22778, 2375, lower.tail=FALSE, log.p=TRUE)

[1] -1587.365

# Number of TBX3/CAPER co-upregulated genes: 446

# Number of TBX3 upregulated genes: 898

# Number of non-TBX3 regulated genes: 24068

# Number of CAPER upregulated genes: 885

> phyper(446, 898, 24068, 885, lower.tail=FALSE)

[1] 0

> phyper(446, 898, 24068, 885, lower.tail=FALSE, log.p=TRUE)

[1] -1016.77

# Number of “transcript regulation” TBX3/CAP co-upregulated genes: 122

# Number of TBX3 “transcript regulation” upregulated genes: 214

# Number of non-TBX3 regulated genes: 24752

# Number of CAPER “transcript regulation” upregulated genes: 248

> phyper(122, 214, 24752, 248, lower.tail=FALSE)

[1] 4.149121e-201

> phyper(122, 214, 24752, 248, lower.tail=FALSE, log.p=TRUE)

[1] -461.3967

# Number of TBX3/CAPER co-downregulated genes: 711

# Number of TBX3 downregulated genes: 1290

# Number of non-TBX3 regulated genes: 23676

# Number of CAPER downregulated genes: 1490

> phyper(771, 1290, 23676, 1490, lower.tail=FALSE)

[1] 0

> phyper(771, 1290, 23676, 1490, lower.tail=FALSE, log.p=TRUE)

[1] -1562.026

# Number of TBX3/CAPER “cell cycle” co-downregulated genes: 101

# Number of TBX3 “cell cycle” downregulated genes: 132

# Number of non-TBX3 regulated genes: 24,834

# Number of CAPER “cell cycle” downregulated genes: 131

> phyper(101, 132, 24834, 131, lower.tail=FALSE)

[1] 1.473818e-228

> phyper(101, 132, 24834, 131, lower.tail=FALSE, log.p=TRUE)

[1] -524.6015

# Number of TBX3/CAP/RAS co-upregulated genes: 48

# Number of TBX3/CAP upregulated genes: 446

# Number of non-TBX3/CAP regulated genes: 24,520

# Number of RAS upregulated genes: 533

> phyper(48, 446, 24520, 533, lower.tail=FALSE)

[1] 4.801644e-21

> phyper(48, 446, 24520, 533, lower.tail=FALSE, log.p=TRUE)

[1] -46.78533

# Number of TBX/CAP/RAS co-downregulated genes: 214

# Number of TBX3/CAP downregulated genes: 711

# Number of non-TBX3/CAP regulated genes: 24,255

# Number of RAS regulated genes: 641

> phyper(214, 711, 24255, 641, lower.tail=FALSE)

[1] 5.664513e-176

> phyper(214, 711, 24255, 641, lower.tail=FALSE, log.p=TRUE)

[1] -403.5208
